# Analysis of Instagram^®^ Posts Referring to Cleft Lip

**DOI:** 10.3390/ijerph17207404

**Published:** 2020-10-12

**Authors:** Magdalena Sycinska-Dziarnowska, Piotr Stepien, Joanna Janiszewska-Olszowska, Katarzyna Grocholewicz, Maciej Jedlinski, Roberta Grassi, Marta Mazur

**Affiliations:** 1 Department of Interdisciplinary Dentistry, Pomeranian Medical University in Szczecin, 70111 Szczecin, Poland; magdadziarnowska@gmail.com (M.S.-D.); katgro@pum.edu.pl (K.G.); maciej.jedlinski@hotmail.com (M.J.); 2 Department of Technology and Education, Koszalin University of Technology, 75453 Koszalin, Poland; piotr.stepien@tu.koszalin.pl; 3 Department of General Dentistry, Pomeranian Medical University in Szczecin, 70111 Szczecin, Poland; 4 Department of Surgical, Medical and Experimental Sciences, University of Sassari, 07100 Sassari, Italy; grassi.roberta93@gmail.com; 5 Department of Oral and Maxillofacial Sciences, Sapienza University of Rome, 00161 Rome, Italy; marta.mazur@uniroma1.it

**Keywords:** cleft palate, internet, social media

## Abstract

*Background*: Social media has become a source of medical information. Cleft lip and palate is a visible congenital anomaly. The aim of the study was to analyze Instagram^®^ posts on the topic of cleft lip. *Methods*: Instagram^®^ posts with “#cleftlip” from March 2014–March 2017 were accessed. Separate lists of expressions (hashtags, meaningful words, words with emojis or emojis alone) were prepared for primary posts and for replies. Thirty expressions statistically most frequent in primary versus secondary posts and 30 in secondary versus primary posts were identified (Group 1) as well as 30 English words or hashtags (Group 2), non-English words or hashtags (Group 3) and emojis (Group 4). The frequencies of expressions were compared (Z-test for the difference of two population proportions). *Results*: There were 34,129 posts, (5427 primary posts and 28,702 replies), containing 62,163 expressions, (35,004 in primary posts). The occurrence of all expressions was 454,162, (225,418 in primary posts and 228,744 in replies). Posts with positive expressions such as “beautiful”, “love”, “cute”, “great”, “awesome” occurred more often than these with negative ones. In replies all emojis were positive. *Conclusions*: Numerous Instagram^®^ posts referring to cleft lip are published and do provoke discussion. People express their solidarity and sympathize with persons affected by cleft.

## 1. Introduction

Instagram^®^ (Facebook, Inc., Menlo Park, CA, USA) is an online photo-sharing application and service, where users may share pictures or videos. It was launched in October 2010 as a free mobile application. On 21 December 2016 it was announced that its community has grown to more than 600 million users and the last 100 million of Instagrammers joined in the past six months [1]. On 26 April 2017 it was announced on Instagram^®^ website that its popularity has grown to 700 million Instagrammers [1].

Since 2012, when 13% people used this service, a significant increase of usage is observed. According to a national survey carried out between 7 March and 4 April 2016 on 1520 adults, about 32% of online adults (e.g., American people who currently use the Internet, according to the same survey it was 86% of the population) or 28% of all adult Americans report using Instagram^®^—roughly the same share as in 2015, when 27% of online adults used the application. Instagram^®^ use was especially high among younger adults; 59% Instagrammers were people between 18–29 year, whereas 8% only were older than 65. According to Pew Research Center women were more likely to use this service (38%) than men (26%). Half of Instagram^®^ users access the platform daily, 35% of them several times a day [2].

Instagram^®^ reflects major social trends, especially among young population. People use Instagram^®^ for communication and entertainment or to express thoughts, moods and feelings. Nowadays, social media play an important role in searching for information by medical caregivers or patients who might look for answers to their questions online [3,4,5,6]. Several papers pertaining to the use of social media in the context of a medical problem (diabetes, Zika virus, arthroplasty) could be found. By interacting with other users, people with medical problems may provide or gain support and share information.

Numerous social media network analyzers are available online. One of them is Netlytic [7]. It allows to automatically summarize and gather data from online conversations found on social media sites [7]. A few questionnaire studies on the use of social media in the context of cleft lip has been published in the recent years [8,9]. No studies based on social media surveillance for cleft lip could be found.

The aim of the study was to analyze the frequency of individual meaningful words, emojis, emoticons or hashtags and to compare their frequency in Instagram^®^ posts and their replies.

## 2. Materials and Methods

### 2.1. Instagram^®^ Surveillance

The analysis of the content of Instagram^®^ posts was initiated by querying the hashtag #cleftlip using the Netlytic service (netylic.org, Toronto, ON, Canada), an open-sourced software. All tagged messages with the #cleftlip hashtag on Instagram^®^ were downloaded and exported to a spreadsheet. The download was started on 17 February 2017; it enabled data collection from Instagram^®^ search every hour until 4 March 2017. The data gathered covered all posts from public profiles published by Instagram^®^. The first captured post was published on 11 March 2014 and the last one on 4 March 2017. Thus, the data contained posts published for almost three years.

The posts collected were divided into primary posts and secondary posts (replies). Primary posts consisted of pictures and their descriptions posted by Instagram^®^ users. Secondary posts were responses to primary posts written by other users or by the author of primary post. Two separate lists of all expressions (hashtags, meaningful words, words combined with emojis or emojis alone) present in the posts were prepared for primary posts and for secondary ones. For the purpose of this article both emojis and emoticons were later stated as emojis. A spreadsheet macro was written to assign each expression the number of occurrences in all primary and secondary posts. Capitalization of letters was ignored as well as dots, commas, exclamation marks and question marks. Then, both lists were grouped into one, containing expressions sorted in descending order by the number of occurrences in primary posts (*x*_1_), and each expression was also assigned the number of occurrences in secondary posts (*x*_2_). The total list consisted of 62,163 expressions.

The proportion ${\hat{p}}_{1}$ of the number of occurrences of each expression in the total number of occurrences of all expressions in primary posts (*n*_1_) was calculated. A similar procedure was applied to secondary posts in order to calculate proportion ${\hat{p}}_{2}$ from *x*_2_ and the total number of occurrences of all expressions in secondary posts (*n*_2_):

(1) $${\hat{p}}_{1}=\frac{x_{1}}{n_{1}}$$

(2) $${\hat{p}}_{2}=\frac{x_{2}}{n_{2}}$$

On the basis of the calculated absolute value of the test statistic z (see the section “Statistical analysis”), 30 expressions statistically most frequent in the primary versus secondary posts were identified and tabularized. Similarly, 30 expressions statistically most frequent in the secondary versus primary posts were identified. All together the 60 expressions have been placed in Table 1 and designated as Group 1 of expressions. The same procedure has been applied to English words or hashtags (Group 2, Table 2), non-English words or hashtags (Group 3, Table 3) and emojis (Group 4, Table 4). There were no emojis statistically more frequent found in primary versus secondary posts, thus Table 4 consists of 30 emojis from secondary posts only. 

Names, surnames, question marks, conjunctions, colons, pronouns and dashes were ignored as well as non-meaningful expressions. Emojis occurring one by one were considered as an independent type of emoji. For example, three hearts that occurred one by one were treated independently, as a more expressive form. Non-English expressions were recognized using available online dictionaries and translators (in most cases by Google Translator). If a non-English expression consisted of several words (e.g., #pormaissorrisossemvergonha), they were separated (#por mais sorrisos sem vergonha) and translated with Google Translator (#for more shameless smile) and then concatenated (#formoreshamelesssmile).

### 2.2. Statistical Analysis

The frequencies of selected expressions in the posts and their replies were compared using a z-test for the difference of two population proportions. The test statistic were applied for expressions meeting the following conditions: $\min\left(n_{1}{\hat{p}}_{1},n_{1}\left(1-{\hat{p}}_{1}\right)\right)\geq 5$ and $\min\left(n_{2}{\hat{p}}_{2},n_{2}\left(1-{\hat{p}}_{2}\right)\right)\geq 5$.

The null hypothesis $H_{0}:p_{1}=p_{2}$ (the proportions in both statistical populations are equal) and the following alternative hypotheses were checked:

- two-tailed $H_{1}:p_{1}\neq p_{2}$ (the proportions in both populations differ);
- one-tailed $H_{1}:p_{1}>p_{2}$ or $H_{1}:p_{1}<p_{2}$ (proportion among the expressions occurring in primary posts was higher than the proportion of expressions occurring in secondary posts and the proportion of expressions occurring in primary posts was lower than the proportion of expressions occurring in secondary posts respectively).

Three tests were made for the following hypotheses:

**Hypothesis** **1.** *H_0_ : p_1_ = p_2_, H_1_ : p_1_ ≠ p_2_*

**Hypothesis** **2.** *H_0_ : p_1_ = p_2_, H_1_ : p_1_ > p_2_*

**Hypothesis** **3.** *H_0_ : p_1_ = p_2_, H_1_ : p_1_ ∈ p_2_*

Test 1 was performed for each expression, test 2 for expressions for which ${\hat{p}}_{1}>{\hat{p}}_{2}$, and test 3 for expressions for which ${\hat{p}}_{1}<{\hat{p}}_{2}$.

For each expression the pooled proportion $\bar{p}$ was calculated:

(3) $$\bar{p}=\frac{x_{1}+x_{2}}{n_{1}+n_{2}}$$

Value of test statistic was calculated as:

(4) $$z=\frac{{\hat{p}}_{1}-{\hat{p}}_{2}}{\sqrt{\bar{p}\left(1-\bar{p}\right)\left(\frac{1}{n_{1}}+\frac{1}{n_{2}}\right)}}$$

The level of statistical significance was set at α = 0.05 and for each test *p*-value was calculated. By comparing the *p*-value with the statistical significance level, it was stated whether there was enough evidence for rejecting H_0_ for H_1_ (*p*-value < α) or not (*p*-value ≥ α). The datasets used and analyzed during the current study are available from the corresponding author on reasonable request.

## 3. Results

The total number of posts downloaded was 34,129 including 5427 primary posts and 28,702 secondary posts. There were 62,163 expressions found in all posts, including 35,004 in primary posts. The total occurrence of all expressions was 454,162, including 225,418 in primary posts and 228,744 in secondary posts. The test statistic conditions have been met for *n*_1_ = 147,323 occurrences of expressions in primary posts and *n*_2_ = 161,270 in secondary posts. As a result, 59,812 expressions (96.22% of total) had to be removed due to statistically unitary character, so 2351 expressions were tested. Finally, there were 4702 tests performed: 2351 for two-tailed alternative hypothesis and 2351 for one-tailed alternative hypotheses. The summary of the number of test cases decisions for the entire population is presented in Table 5. 1337 expressions were more frequent in primary posts and 1014 expressions were more frequent in secondary posts. As many as 1148 expressions were statistically significantly more frequent in primary or in secondary posts. However, 57.64% of the expressions were as often found in primary as in secondary posts, and 42.36% of expressions showed statistically significantly different frequencies. Out of the expressions that were more frequent in primary than in secondary posts in 51.53% there was no statistically significant dominance over the frequency of occurrences in replies, and 48.47% showed a statistically significantly higher rate of occurrence. Out of the expressions that occurred more frequently in secondary posts than in primary posts, in 50.69% cases there was no statistically significant dominance over the incidence of primary posts, and 49.31% had a statistically significant higher incidence.

It is seen that posts with expressions associated with positive emotions such as beautiful, love, cute, great, awesome occurred more often than with negative ones. In primary posts hashtags were much more frequent than in secondary posts. Among 30 expressions with the most significant difference in frequency of occurrences from the primary posts there were seven words that were not hashtags, for example: lip, cleft and surgery—so words related to the subject of our study. Hashtags used in the posts, help linking the photos and messages up to other subject on Instagram^®^ featuring the same topic.

In the first group of expressions in primary posts dominated hashtags and words associated with cleft lip problem. What was surprising there were hashtags related to animals for example #dog or #puppy. In the same group in secondary posts, expressions with positive meaning were overwhelming.

Among non-English words or hashtags a domination of Portuguese and Spanish languages was evident. Also Indonesian language was frequent especially among non-English words from secondary posts.

In group four, what was very surprising, no emojis were found that appeared more frequently in primary posts. The emojis appearing more frequently in secondary posts versus primary posts were the very popular ones, expressing emotions (heart, smile). In replies all emojis were highly positive.

## 4. Discussion

This is the first study of Instagram^®^ posts dealing with the subject of cleft lip. The present study is based on the largest number of Instagram^®^ posts on a medical problem of all papers found. In the study by Fung et al. [10] 616 Instagram^®^ posts with hashtag #zikavirus were manually coded. Karimkhani et al. [11] analyzed 50 newest posts referring to dermatology. According to thematic content Pila et al. [12] analyzed a sample of 600 Instagram^®^ posts with hashtag #cheatmeal. Another study of 649 Instagram^®^ posts on total knee arthroplasty and 638 posts on total hip arthroplasty was conducted at the Department of Orthopedic Surgery in Cleveland and Houston [6]. Tiggemann and Zaccardo [13] were looking for fitspiration hashtag on Instagram^®^ regarding to body type and activity. A group of the first 600 posts with #fitspiration hashtag were coded for textual and photo content. Yi-Frazier et al. [14] asked twenty teenagers 14 to 18 years old with diabetes type 1 to use Instagram^®^ and post photos on diabetes-related themes. Twelve participants were highly engaged; the whole study lasted for three consecutive weeks. In the study by Chung et al. [15] 16 women were interviewed who consistently shared and recorded on Instagram^®^ what they had eaten. Another study gathered 476 social media posts tagged with #fitspo among the four platforms. Relevant 415 of 476 posts (87.2%) were analyzed. The majority of posts were accessed from Instagram^®^ (360/415, 86.8%) [16].

Medical problems analyzed by previous studies were Zika virus, skin problems, problems with diabetes and in more detail concerning care of diabetes foot [3,5,10,11,17]. Very popular subjects concerning social media were associated with diet, fitness or nutrition [12,13].

Referring to orthodontics, three studies could be found, one—pertaining to patient experience to orthodontic brackets versus Invisalign^®^ [18], another one was a qualitative analysis of orthodontic-related posts on Twitter [19] the last one showed how social media improve knowledge among patients with fixed orthodontic appliance [4].

In the present study, hashtags dominated in primary versus secondary posts. It may be explained by the fact that use of hashtags helps the author of a message to link post with the group of desired subjects.

Pew Research stated that Instagram^®^ is very popular among non-white users. According to this demographic statistics in 2014 Hispanic origin people represent 34% of Instagram^®^ online adult users in the United States of America [20]. This is visible in our study among non-English words or hashtags group (Table 4) represented most often by Spanish and Portuguese languages.

The statistically significant difference in the occurrence of emoji in secondary versus primary posts indicates that replies gave positive responses or comments. This means that many individuals expressed their solidarity and sympathized with persons affected by cleft. The authors find this aspect as very optimistic.

What was remarkable during the review was the fact that there were posts pertained to animals (dogs, cats and a post on a squirrel). This shows that owners of animals with cleft problem also post on Instagram^®^.

It is worth noticing that the number of replies (28,702) was much higher than of the primary posts (5427). This indicates a great interest in the posts concerning cleft lip. No comparison of primary versus secondary posts that might be used for comparison could be found in other studies analyzing social media.

From the fact that for primary posts, the total occurrence of all expressions was 225,418 (in 5427 posts) and for secondary posts 228,744 (in 28,702 posts), we may assume that the replies were shorter. The possible explanation could be that the primary posts contained detailed descriptions and the replies were spontaneous (often short and emotional) reactions to them.

Personalized medicine may create optimal treatment for the group of patients with cleft lip. Each cleft is unique in terms of its morphology. Every individual affected may suffer from different medical and psychological problems.

It is evident that people affected by a cleft interact with one another via Instagram^®^. As social media become frequently used as a source of medical information, professionals should be aware of content available through Instagram^®^ and consider using it as a means to provide health education. In the future, a more detailed surveillance of Instagram^®^ posts with #cleftlip hashtag may help us better understand motivation, experiences and expectations of patients with clefts. In this way we may provide more accurate interdisciplinary and holistic treatment for affected persons.

## 5. Conclusions

(1) Numerous Instagram^®^ posts referring to cleft lip are published and provoke discussion.
(2) In Instagram^®^ posts two groups of meaningful expressions can be identified: one that appear more frequently in primary posts than in secondary posts and the other appearing more often in replies than in primary posts.
(3) Expressions that occur more frequently in secondary posts than in primary ones do not contain offensive words, they are positive. People express their solidarity and sympathize with persons affected by cleft.
(4) Hashtags occur more frequently in primary posts than in replies.

## Figures and Tables

**Table 1:** Group one—expressions with the most statistically significant difference in the frequency of occurrence.

Nº	Expressions Dominating in Primary Posts				Expressions Dominating in Secondary Posts			
	Expression	$x_{1}$	$x_{2}$	abs(z)	Expression	$x_{1}$	$x_{2}$	abs(z)
1	#cleftlip	4110	681	53.136	beautiful	173	1647	32.753
2	#cleftpalate	1662	359	31.150	love	442	1791	26.535
3	#cleftstrong	1459	425	25.889	thank	158	1172	26.239
4	#cleftlipandpalate	572	55	21.823	cute	65	848	24.609
5	#cleft	756	209	19.062	😍	104	835	22.528
6	#1in700	383	29	18.389	❤	84	630	19.268
7	cleft	1254	575	17.881	great	124	701	18.835
8	#cleftcutie	768	247	17.841	nice	18	439	18.760
9	lip	907	344	17.570	awesome	34	478	18.634
10	#cleftproud	563	168	15.867	thanks	70	524	17.561
11	#cleftie	284	25	15.552	lindo	14	373	17.389
12	#smile	303	39	15.135	gorgeous	14	370	17.310
13	#dogs	167	6	12.852	bless	11	360	17.277
14	#puppy	184	13	12.835	:)	36	411	16.811
15	#love	281	60	12.823	adorable	38	416	16.807
16	#cleftbabies	155	5	12.446	sweet	105	568	16.710
17	today	304	80	12.337	wow	5	319	16.657
18	#beautiful	150	5	12.225	😍😍😍	6	301	16.069
19	#labioleporino	218	39	11.907	😘	16	323	15.867
20	mission	155	12	11.665	precious	14	292	15.125
21	#plasticsurgery	208	38	11.564	😊	34	326	14.556
22	#dog	134	7	11.246	god	87	435	14.226
23	#selfie	127	5	11.152	😍😍	9	238	13.880
24	children	235	63	10.760	amazing	171	584	13.820
25	#surgery	159	23	10.705	good	155	552	13.759
26	#squishyfacecrew	148	21	10.370	handsome	34	292	13.494
27	surgery	507	264	10.029	cutie	31	283	13.441
28	new	299	115	9.980	👍	9	222	13.346
29	#charity	157	30	9.918	cool	18	224	12.557
30	#babiesofinstagram	115	10	9.909	looks	59	306	12.085

$x_{1}$—number of occurrences in primary posts, $x_{2}$—number of occurrences in secondary posts, abs(z)—absolute value of test statistic.

**Table 2:** Group two—English words or hashtags with the most statistically significant difference in the frequency of occurrence.

Nº	English Words or Hashtags Dominating in Primary Posts				English Words or Hashtags Dominating in Secondary Posts			
	Word or Hashtag	$x_{1}$	$x_{2}$	abs(z)	Word or Hashtag	$x_{1}$	$x_{2}$	abs(z)
1	#cleftlip	4110	681	53.136	beautiful	173	1647	32.753
2	#cleftpalate	1662	359	31.150	love	442	1791	26.535
3	#cleftstrong	1459	425	25.889	thank	158	1172	26.239
4	#cleftlipandpalate	572	55	21.823	cute	65	848	24.609
5	#cleft	756	209	19.062	great	124	701	18.835
6	#1in700	383	29	18.389	nice	18	439	18.760
7	cleft	1254	575	17.881	awesome	34	478	18.634
8	#cleftcutie	768	247	17.841	thanks	70	524	17.561
9	lip	907	344	17.570	gorgeous	14	370	17.310
10	#cleftproud	563	168	15.867	bless	11	360	17.277
11	#cleftie	284	25	15.552	adorable	38	416	16.807
12	#smile	303	39	15.135	sweet	105	568	16.710
13	#dogs	167	6	12.852	wow	5	319	16.657
14	#puppy	184	13	12.835	precious	14	292	15.125
15	#love	281	60	12.823	god	87	435	14.226
16	#cleftbabies	155	5	12.446	amazing	171	584	13.820
17	today	304	80	12.337	good	155	552	13.759
18	#beautiful	150	5	12.225	handsome	34	292	13.494
19	mission	155	12	11.665	cutie	31	283	13.441
20	#plasticsurgery	208	38	11.564	cool	18	224	12.557
21	#dog	134	7	11.246	looks	59	306	12.085
22	#selfie	127	5	11.152	luck	6	150	10.979
23	children	235	63	10.760	lovely	16	168	10.607
24	#surgery	159	23	10.705	like	296	652	10.197
25	#squishyfacecrew	148	21	10.370	job	29	189	10.183
26	surgery	507	264	10.029	prayers	20	164	10.016
27	new	299	115	9.980	happy	277	611	9.886
28	#charity	157	30	9.918	super	36	197	9.871
29	#babiesofinstagram	115	10	9.909	hi	10	125	9.384
30	#happy	114	10	9.855	lol	26	162	9.311

$x_{1}$—number of occurrences in primary posts, $x_{2}$—number of occurrences in secondary posts, abs(z)—absolute value of test statistic.

**Table 3:** Group three—non-English words or hashtags with the most statistically significant difference in the frequency of occurrence.

Nº	Non-English Words or Hashtags Dominating in Primary Posts					
	Word or Hashtag	Translation	Language	$x_{1}$	$x_{2}$	abs(z)
1	#labioleporino	#cleftlip	Spanish	218	39	11.907
2	#fissuralabiopalatina	#cleftpalate	Spanish	74	9	7.555
3	#maternidade	maternity	Portuguese	65	6	7.391
4	#maedeprimeiraviagem	#firsttimemom	Portuguese	52	7	6.213
5	idag	today	Swedish	54	9	6.035
6	#fissuradapelacecilia	#ceciliasfissure	Portuguese	50	9	5.691
7	#pormaissorrisosemvergonha	#formoreshamelesssmile	Portuguese	39	9	4.648
8	hoje	today	Portuguese	49	17	4.311
9	blev	became	Danish	31	7	4.176
10	#abogoiás	#imfromgoias	Portuguese/Spanish	37	12	3.892
11	bibir	lip	Indonesian	34	11	3.736
12	#maedemenina	#motherofgirl	Portuguese	30	9	3.649
13	labial	lip	Spanish	24	6	3.538
14	pappa	dad	Swedish	22	5	3.510
15	dudak	lip	Turkish	38	15	3.492
16	mamãe	mom	Portuguese	52	25	3.478
17	operasi	operation	Indonesian	45	21	3.325
18	bayi	baby	Indonesian	28	10	3.202
19	vamos	come on	Spanish	25	9	3.011
20	paladar	palate	Spanish	25	9	3.011
21	pacientes	patients	Spanish	20	6	2.979
22	fick	got	Swedish	30	13	2.892
23	hendido	cleft	Spanish	21	7	2.888
24	akan	will	Indonesian	30	14	2.715
25	dias	days	Portuguese	27	12	2.687
26	semanas	week	Portuguese	17	6	2.513
27	hari	day	Indonesian	27	13	2.502
28	labio	lip	Spanish	36	20	2.479
29	fissura	fissure	Portuguese	28	14	2.456
30	#associacaoreface	#faceassociation	Portuguese	60	41	2.348
1	lindo	pretty	Portuguese/Spanish	14	373	17.389
2	linda	pretty	Portuguese/Spanish	11	160	10.817
3	gracias	thank you	Spanish	15	141	9.536
4	deus	God	Portuguese	41	193	9.258
5	sehat	healthy	Indonesian	6	67	6.761
6	muito	much	Portuguese	78	197	6.436
7	coisa	thing	Portuguese	9	57	5.547
8	mais	more	Portuguese	117	222	4.878
9	amor	love	Portuguese/Spanish	26	80	4.785
10	terus	continue	Indonesian	6	37	4.436
11	anak	child	Indonesian	34	88	4.395
12	buat	create	Indonesian	7	33	3.829
13	selalu	always	Indonesian	9	37	3.826
14	certo	of course	Italian	8	31	3.404
15	mesmo	same	Portuguese	17	47	3.392
16	est	east	French/Italian	9	32	3.306
17	bara	only	Swedish	5	24	3.288
18	sempre	all time	Italian	33	71	3.269
19	obrigada	thanks	Portuguese	18	44	2.949
20	kasih	love	Indonesian	8	26	2.826
21	gran	great	Spanish	5	20	2.777
22	mejor	best	Spanish	8	25	2.703
23	mau	want	Indonesian	11	30	2.681
24	skrg	now	Indonesian	6	21	2.655
25	cara	dear	Italian	5	19	2.639
26	quero	want	Portuguese	5	19	2.639
27	nasceu	was born	Portuguese	12	31	2.604
28	tenho	I have	Portuguese	10	27	2.523
29	nya	new	Swedish	39	70	2.500
30	sama	same	Indonesian	19	41	2.493

$x_{1}$—number of occurrences in primary posts, $x_{2}$—number of occurrences in secondary posts, abs(z)—absolute value of test statistic.

**Table 4:** Group four—emojis with the most statistically significant difference in the frequency of occurrence.

Nº	Emojis Dominating in Secondary Posts			
	Emoji	$x_{1}$	$x_{2}$	abs(z)
1	😍	104	835	22.528
2	❤	84	630	19.268
3	:)	36	411	16.811
4	😍😍😍	6	301	16.069
5	😘	16	323	15.867
6	😊	34	326	14.556
7	😍😍	9	238	13.880
8	👍	9	222	13.346
9	💕	52	198	8.531
10	😃	6	97	8.518
11	😄	5	93	8.452
12	❤❤	10	102	8.225
13	👌	10	94	7.785
14	☺	5	70	7.122
15	💖	5	67	6.931
16	😂😂😂	13	78	6.390
17	😉	10	70	6.311
18	💗	15	81	6.301
19	💜	9	67	6.266
20	😀	11	71	6.224
21	💙	65	171	6.214
22	🙌	13	72	5.990
23	☺	6	51	5.625
24	💕💕	5	47	5.505
25	😁	16	71	5.481
26	😂😂	17	70	5.267
27	💛	6	44	5.060
28	♡	13	57	4.886
29	😎	7	41	4.599
30	😂	5	33	4.268

$x_{1}$—number of occurrences in primary posts, $x_{2}$—number of occurrences in secondary posts, abs(z)—absolute value of test statistic.

**Table 5:** Summary of test results.

$H_{0}:p_{1}=p_{2}$ $H_{1}:p_{1}\neq p_{2}$			$H_{0}:p_{1}=p_{2}$ $H_{1}:p_{1}>p_{2}$			$H_{0}:p_{1}=p_{2}$ $H_{1}:p_{1}<p_{2}$		
NE	IE	Total	NE	IE	Total	NE	IE	Total
1355	996	2351	689	648	1337	514	500	1014
57.64%	42.36%	100.00%	51.53%	48.47%	100.00%	50.69%	49.31%	100.00%

IE—there is enough evidence to reject H0, NE—there is not enough evidence to reject H0.
